# An Autoinhibitory Tyrosine Motif in the Cell-Cycle-Regulated Nek7 Kinase Is Released through Binding of Nek9

**DOI:** 10.1016/j.molcel.2009.09.038

**Published:** 2009-11-25

**Authors:** Mark W. Richards, Laura O'Regan, Corine Mas-Droux, Joelle M.Y. Blot, Jack Cheung, Swen Hoelder, Andrew M. Fry, Richard Bayliss

**Affiliations:** 1 Section of Structural Biology, Institute of Cancer Research, Chester Beatty Laboratories, 237 Fulham Road, London SW3 6JB, UK; 2 Department of Biochemistry, University of Leicester, Leicester LE1 9HN, UK; 3 Cancer Research UK Centre for Cancer Therapeutics, The Institute of Cancer Research, Haddow Laboratories, 15 Cotswold Road, Sutton, Surrey SM2 5NG, UK

**Keywords:** SIGNALING, PROTEINS, CELLCYCLE

## Abstract

Mitosis is controlled by multiple protein kinases, many of which are abnormally expressed in human cancers. Nek2, Nek6, Nek7, and Nek9 are NIMA-related kinases essential for proper mitotic progression. We determined the atomic structure of Nek7 and discovered an autoinhibited conformation that suggests a regulatory mechanism not previously described in kinases. Additionally, Nek2 adopts the same conformation when bound to a drug-like molecule. In both structures, a tyrosine side chain points into the active site, interacts with the activation loop, and blocks the αC helix. Tyrosine mutants of Nek7 and the related kinase Nek6 are constitutively active. The activity of Nek6 and Nek7, but not the tyrosine mutant, is increased by interaction with the Nek9 noncatalytic C-terminal domain, suggesting a mechanism in which the tyrosine is released from its autoinhibitory position. The autoinhibitory conformation is common to three Neks and provides a potential target for selective kinase inhibitors.

## Introduction

Entry into mitosis and assembly of the bipolar mitotic spindle are regulated by several serine/threonine protein kinases, including members of the cyclin-dependent kinase (Cdk), Polo-like kinase (Plk), Aurora, and NIMA-related kinase (Nek) families (Barr et al., 2004; Carmena and Earnshaw, 2003; Nigg, 2001; O'Regan et al., 2007). NIMA was discovered in *Aspergillus*, where it is essential for mitotic entry and is capable of driving cells into mitosis from any point in the cell cycle (Morris, 1975; Osmani et al., 1988). The Nek family comprises 11 members in humans. The expansion of the Nek family is partly due to its extension into cilia function, as mutations in Nek1 and Nek8 are causative for mouse models of ciliopathies (Quarmby and Mahjoub, 2005). Nek2, -6, -7, and -9 have mitotic functions, although none of them is a true functional homolog of *Aspergillus* NIMA. Nek2 has a clear role in the separation of duplicated centrosomes at mitotic onset (Fry, 2002). Less is known about Nek6, -7, and -9, although they are all essential for proper mitotic spindle assembly (Kim et al., 2007; O'Regan and Fry, 2009; Roig et al., 2002; Yin et al., 2003; Yissachar et al., 2006).

Nek6 and Nek7 are the smallest members of the Nek family, comprising only a catalytic domain with a 30–40 amino acid N-terminal extension (Kandli et al., 2000). In amino acid sequence, the kinases are 86% identical within the catalytic domain and are 100% identical in residues that line the ATP-binding pocket. The N-terminal extensions are not conserved, and it has been suggested that they may play a role in differential regulation of the kinases (Minoguchi et al., 2003). In mitosis, both kinases are phosphorylated and exhibit much higher activity than in interphase (O'Regan and Fry, 2009). Overexpression of kinase-dead protein or RNAi results in mitotic spindle defects, increased mitotic index, increased multinuclear cells, and increased apoptosis (Kim et al., 2007; O'Regan and Fry, 2009; Yissachar et al., 2006). There are currently no known substrates of Nek7, but Eg5, a microtubule motor protein essential for mitotic spindle assembly, has recently been identified as a substrate of Nek6 (Rapley et al., 2008). The fact that RNAi depletion of either kinase leads to mitotic progression defects indicates that they are nonredundant, although they may yet function in the same pathway. This pathway almost certainly involves Nek9, and it has been proposed that Nek9 is the upstream kinase responsible for activating Nek6 and Nek7 in mitosis through phosphorylation of residues within their activation loops (Belham et al., 2003). Nek9 consists of an N-terminal catalytic domain, a central RCC1-like domain, and a C-terminal domain (CTD) containing a coiled-coil motif. Interestingly, Nek6 and Nek7 interact strongly with Nek9 in a region far from its catalytic domain, adjacent to its C-terminal coiled-coil motif (Roig et al., 2002). The association between Nek6 and Nek9 is much more prominent during mitosis (Rapley et al., 2008). Nek9 is itself activated during mitosis, and the phosphorylated Nek9 is strongly localized to spindle poles (Roig et al., 2005). Thus, Nek6, -7, and -9 form a network that regulates robust mitotic spindle assembly.

The first structural studies of protein kinases identified the residues which must be precisely positioned for catalysis and the conserved motifs within which they lie (reviewed by Johnson et al., 1996). For example, a lysine holds the phosphates of ATP in position and is in turn held in place through an interaction with a glutamic acid on helix αC. Additionally, an aspartic acid within the conserved DFG (aspartic acid, phenylalanine, glycine) or DLG (aspartic acid, leucine, glycine) motif activates a divalent cation associated with the γ-phosphate of ATP. The DFG/DLG motif lies at the N terminus of the activation loop, which in many kinases must be phosphorylated in order to form an ordered substrate-binding platform. Recently, a set of four residues within the catalytic domain that form a hydrophobic spine has been identified as another conserved feature of the active conformation (Kornev et al., 2006). These residues include the phenylalanine/leucine of the DFG/DLG motif, a hydrophobic residue at the N-terminal end of strand β4, a hydrophobic residue on the αC helix, and a tyrosine/histidine residue in the C lobe. By contrast with their very similar active conformations, kinase structures display a diversity of inactive conformations that reflects the variety of regulatory mechanisms. Nek2 is the only NIMA-related kinase for which structures have been reported (Rellos et al., 2007; Westwood et al., 2009). These structures show the apo-form and three different ligand-bound complexes and particularly highlight the ligand dependency of the activation loop conformation.

The mechanisms of regulation of Nek6, -7, and -9 kinases are currently unknown, and resolving this pathway would shed light on early mitotic events. To make the first step toward this goal, we determined the structure of human Nek7 using X-ray crystallography and investigated its regulation by the noncatalytic CTD of Nek9. We discovered that Nek7 is maintained in a catalytically inactive form by an autoinhibitory motif that, to our knowledge, has not been previously described. A tyrosine residue points into the active site, forming an H-bond with the DLG motif and blocking an inward, active conformation of the αC helix. We additionally show that a similar conformation of the equivalent tyrosine residue is induced in Nek2 bound to a drug-like molecule. This tyrosine residue is not a unique feature of Nek7 and Nek2 but is found at the same position in over 10% of human kinases. In addition, the other residues involved in the autoinhibitory conformation are highly conserved features of kinase catalytic domains. Thus, we propose that a similar motif may play a role in the regulation of other kinases.

## Results

### Crystal Structure of Nek7 Reveals an Autoinhibited Conformation Suggesting a Mechanism for Its Regulation

**Figure 1:**
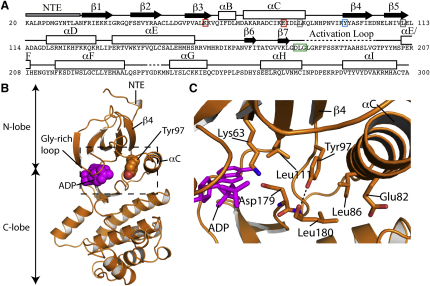
The Crystal Structure of Nek7 Shows an Autoinhibited Conformation that Suggests a Regulatory Mechanism Not Previously Described in Kinases (A) Secondary structure and key residues mapped onto the protein sequence of Nek7. Disordered regions are marked with a dashed line, and ordered regions are marked with a solid line, black arrow, or white rectangle to indicate random coil, β strand, and α helix respectively. Lys63 and Glu82 are marked with red boxes, Leu86 and Leu111 with gray boxes, Tyr97 with a cyan box, and the DLG motif with a green box. (B) Overview of ADP-bound Nek7 structure in cartoon representation. The side chain of Tyr97 and the ADP ligand (magenta) are shown as spheres. The dashed box indicates the region magnified in (C). (C) Cartoon and stick representation of Tyr97 and its surrounding environment. The H-bond between Tyr97 and the backbone of Leu180 is shown as a dashed line.

We crystallized the apo-form, full-length (35 kDa) human Nek7 protein and determined the structure to 2.1 Å. We also soaked the apo-form crystals with ADP and determined the ADP-bound structure to 2.3 Å resolution. Both structures have excellent refinement and geometry statistics (Table 1). The protein structures exhibit the canonical bilobal fold and are virtually identical. Residues 20–300 were modeled, with the exception of the disordered activation loop (residues 181–198) and a small loop (residues 237–240) (Figure 1A). The Gly-rich loop was more ordered in the ADP-bound structure, consistent with an interaction with the ADP ligand. The overall conformation is that of an inactive kinase with the αC helix, DLG motif, and activation loop displaced from the usual configuration observed in active enzymes (Figures 1B and 1C).

**Figure 2:**
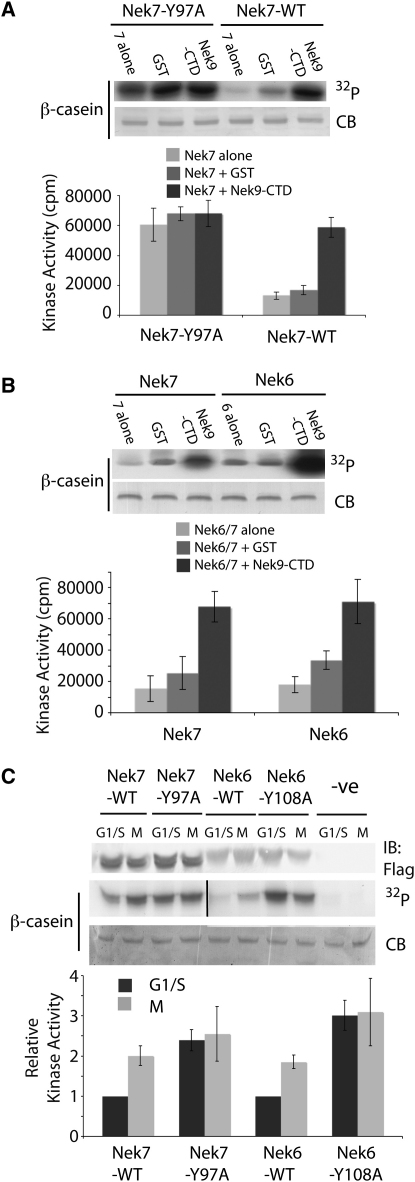
Nek9-CTD or Mutations at Tyr97^Nek7^ and Tyr108^Nek6^ Increase Kinase Activity (A) In vitro kinase activity assay shows the relative activity of wild-type Nek7 (Nek7-WT) and the Tyr97-to-Ala mutant (Nek7-Y97A) in the presence or absence of GST-tagged Nek9 CTD (Nek9-CTD) or control GST. The upper panels show an autoradiograph (^32^P) of a Coomassie blue-stained gel (CB) in a single assay. The histograms in the lower panel show the average of three independent in vitro kinase assay experiments, and the error bars show the standard deviation. (B) In vitro kinase activity assay shows the activity of Nek7 and Nek6 in the presence or absence of GST-tagged Nek9 CTD (Nek9-CTD) or control GST. The upper panels show an autoradiograph (^32^P) of a CB-stained gel in a single assay. The histograms in the lower panel show the average of three independent in vitro kinase assay experiments, and the error bars show the standard deviation. (C) Shown is cell-cycle-specific kinase activity of Flag-tagged, wild-type Nek7 and Nek6 (Nek7-WT, Nek6-WT), Tyr97/Tyr108-to-Ala mutant (Nek7-Y97A, Nek6-Y108A), or untransfected control (-ve) immunoprecipitated from 293 cell lysates synchronized in G1/S or M phase. The upper panel shows an immunoblot of the kinase protein levels and the result of a single assay as an autoradiograph (^32^P). The histograms in the lower panel show the average of three independent kinase assay experiments in which the amount of β-casein phosphorylation was normalized for the amount of protein immunoprecipitated. Error bars show standard deviation.

The most striking feature of the structure is the position of Tyr97, which juts from between β4 and αC into the active site of Nek7 and forms an H-bond between its hydroxyl and the backbone amide of Leu180 within the DLG motif (Figures 1B and 1C). The DLG motif is in an inactive conformation, perhaps held by this H-bond. The aromatic ring of Tyr97 is sandwiched between the gatekeeper residue Leu111 and Leu86. In an active kinase structure, the two leucine residues would pack together, forming part of the hydrophobic core of the kinase and enabling the formation of a salt bridge between Lys63 and Glu82, two residues that are highly conserved in kinases and which must be properly positioned for catalysis. In the Nek7 structure, αC is positioned outwards and twisted away so that the salt bridge between Lys63 and Glu82 cannot form (Figure 1C). We predicted that the position of Tyr97 might be autoinhibitory, and tested this hypothesis by producing a Tyr97-to-Ala mutant (Nek7-Y97A) and assaying its in vitro activity relative to wild-type. We found that Nek7-Y97A was approximately 5-fold more active than the wild-type kinase, showing that this residue has an autoinhibitory function (Figure 2A). In the wild-type protein, this residue must be moved from its inhibitory position to enable full catalytic activity.

### Autoinhibition of Nek7 Is Released by the Noncatalytic Domain of Nek9

Protein kinases are commonly activated by phosphorylation of their activation loops, but a few kinases are additionally activated by protein partners. The activation of CDKs by their cognate cyclins is the archetype, and the activation of Aurora-A by TPX2 is a further example (Bayliss et al., 2003; Pavletich, 1999). In these cases, binding partners interact with helix αC and strand β4, causing conformational shifts in the catalytic domain. The activation of Nek7 through a means other than phosphorylation has not been previously published. Nonetheless, because Tyr97 lies between β4 and αC, we postulated that the movement of Tyr97 might be similarly regulated through the binding of a partner protein. Nek9 is the only known binding partner for Nek7 and is a potential upstream kinase for Nek7, although the interaction between the two proteins is mediated via the noncatalytic Nek9-CTD. We found that Nek9-CTD increased the activity of Nek7-WT to a level similar to that observed for Nek7-Y97A (Figure 2A). Strikingly, Nek9-CTD did not significantly increase the activity of Nek7-Y97A, suggesting that the mechanism of Nek7 activation occurs through release of Tyr97 autoinhibition.

The catalytic domains of Nek6 and Nek7 are very similar, which led us to investigate whether Nek6 is regulated by the same mechanism. Nek9-CTD stimulated Nek6 activity by the same factor as it stimulated Nek7, suggesting that Nek6 may be subject to autoinhibition by Tyr108^Nek6^, the equivalent residue to Tyr97^Nek7^ (Figure 2B). We next investigated the effect of the Y97A^Nek7^ and Y108A^Nek6^ mutations on the cell-cycle-dependent activity of the two proteins by the expression of Flag-tagged proteins in 293 cells (Figure 2C). As previously observed, recombinant protein levels were relatively constant through the cell cycle, and the wild-type kinases showed a peak of activity during mitosis (O'Regan and Fry, 2009). By contrast, Nek7-Y97A and Nek6-Y108A exhibited similar, high levels of activity in G1/S or M phase. The loss of cell-cycle control of Nek7-Y97A activity was deleterious to the 293 cells, which exhibited increased cell death (30%) compared to untransfected or Nek7-WT transfected cells (both 5%). Thus the repression of Nek7 activity in interphase through Tyr97 autoinhibition is a crucial regulatory mechanism. These findings demonstrate the physiological significance of the autoinhibition mechanism observed in the crystal structure and show that the mechanism is conserved between Nek6 and Nek7. This prompted us to investigate the details of why this conformation of Tyr97 in Nek7 is inhibitory.

### Structural Determinants of Autoinhibition by Tyr97

Of the over 500 human kinases, more than 50 possess a tyrosine residue at the equivalent position to Tyr97, including 8 out of 11 human Neks (see Figure S1 available online). Structures of 11 of these kinases or their homologs have been deposited in the Protein Data Bank (PDB). There is only one example in which the tyrosine residue points down into the active site similar to Nek7, in one of the four chains of OSR1 kinase (PDB code 2VWI; Villa et al., 2008). The significance of this structure is not clear, because much of the N lobe is disordered in this chain, including most of the αC. Indeed, the authors of the paper reporting the structure did not comment on this feature. All other kinase structures, including the other three chains of OSR1 kinase, exhibit a rotamer that positions the tyrosine side chain on the surface parallel to the β4 strand (for example, Nek2-ADP in Figure 5A). We shall refer to these two conformations as Tyr-down (i.e., down into the active site) for the Nek7 and Tyr-up (i.e., up on the surface of the kinase) for the position observed in other kinases. Since there is no reported reference of a kinase exhibiting the Tyr-down conformation, we compared the structure of Nek7 with similar kinases to find an explanation for the autoinhibitory function of Tyr97^Nek7^.

**Figure 3:**
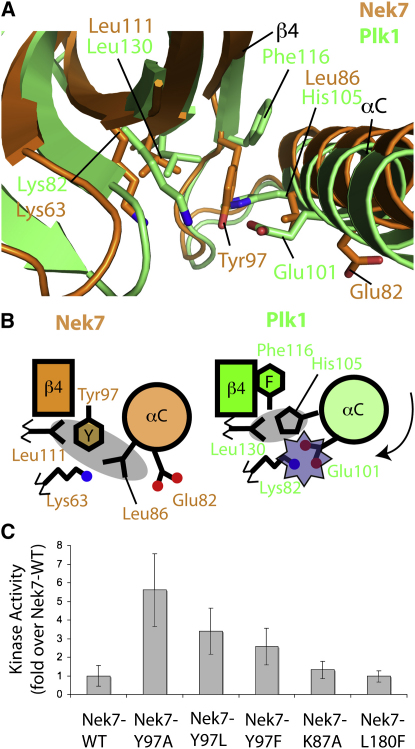
Comparison of the Nek7 Structure with that of an Active Kinase Suggests that Tyr97 Blocks the Active Position of the αC Helix (A) Superposition of Nek7 (orange) centered on Tyr97 with Plk1 in an active conformation (green, PDB code 2V5Q) shows the key residues that must move for Nek7 to adopt an active conformation. (B) Schematic representation of the differences between Nek7 (left, orange) and Plk1 (green, right) shown in a similar view to that in (A). The two secondary structure elements, strand β4 and helix αC, are shown as a rectangle and circle, respectively. Equivalent residues are shown as black lines, hydrophobic interactions are shown as transparent gray ellipses, and an electrostatic interaction that correctly positions the catalytic Lys residue in the active Plk1 structure is shown as a transparent purple star. (C) In vitro kinase activity assay shows the relative activity of wild-type Nek7 (Nek7-WT), the activating Y97A mutant (Nek7-Y97A), and a series of mutants designed to investigate the structural determinants for Tyr97 autoinhibition. The histograms show the average of four independent experiments, and the error bars show the standard deviation.

Using DALI, we identified Nek2 (Z score 26.9, 2.6 Å root-mean-square deviation [rmsd], 36% identity) and Plk1 structures (Z score 26.8, 2.4 Å rmsd, 24% identity) as most similar to the Nek7 structure. We superposed Nek7 with Plk1 in its active conformation to help visualize the conformational changes that would be required for an active Nek7 (Figure 3A), and we have summarized these changes in a schematic diagram (Figure 3B). By contrast with Nek7, which exhibits an inactive, outward position of αC, in Plk1 the αC is inward and in the correct orientation so that Lys82^Plk1^ and Glu101^Plk1^ form a salt bridge, unlike the equivalent residues Lys63^Nek7^ and Glu82^Nek7^. His105^Plk1^ on αC sits close to the Leu130^Plk1^ gatekeeper, in the equivalent pocket to that occupied by Tyr97^Nek7^. The equivalent residue to Tyr97^Nek7^ is Phe116^Plk1^, which adopts a position analogous to the Tyr-up conformation. These features of the active Plk1 structure are highly conserved among other active kinase structures, as are the residues involved (Figure S1). In particular, the need to form a salt bridge between residues equivalent to Lys63^Nek7^ and Glu82^Nek7^, and the position of residues that form a hydrophobic spine including residues equivalent to Leu86^Nek7^ and Tyr97^Nek7^, is crucial for catalytic activity (Johnson et al., 1996; Kornev et al., 2006). Thus, the Tyr-down rotamer is incompatible with an active conformation, because it disrupts the hydrophobic spine and blocks the contact between the gatekeeper residue and the hydrophobic residue on αC. This prevents αC from adopting the inward conformation, which prevents the contact between Lys63^Nek7^ and Glu82^Nek7^. It is likely that mutation of Tyr97^Nek7^ to Ala relieves this inhibition by removing the blockage, thus allowing the movement of αC into an inward, active position that requires Leu86^Nek7^ to contact Leu111^Nek7^. It is possible that the H-bond between Tyr97 and the DLG motif also contributes to inhibition by holding the activation loop in an inactive conformation.

We produced a series of point mutations to test the requirement for a tyrosine residue at position 97 (Figure 3C). Nek7-Y97F and Nek7-Y97L were designed to test the contribution to inhibition of the H-bond with the DLG motif. Leu and Phe are the two most common alternatives to tyrosine in this position in human kinases, found in 50% and 13% of kinases, respectively. These mutants showed activity intermediate between that of Nek7-WT and Nek7-Y97A, suggesting that the H-bond with Leu180^Nek7^ is an important determinant of autoinhibition. The divergent DLG motif does not seem to be a factor, because the mutant in which this was altered to the more common DFG sequence, Nek7-L180F, showed similar activity to Nek7-WT. The creation of a surface pocket in the Nek7-K87A mutant also has no significant effect. The limited effect of this mutant suggests that the preference for the Tyr-down conformation is not simply an effect of lack of space for the side chain on the protein surface, and is consistent with the idea that the tyrosine is held down by the H-bond with the Leu180^Nek7^ mainchain and hydrophobic packing against Leu111^Nek7^ and Leu86^Nek7^ side chains.

### CCT241950 Induces the Tyr-Down Conformation in Nek2

**Figure 4:**
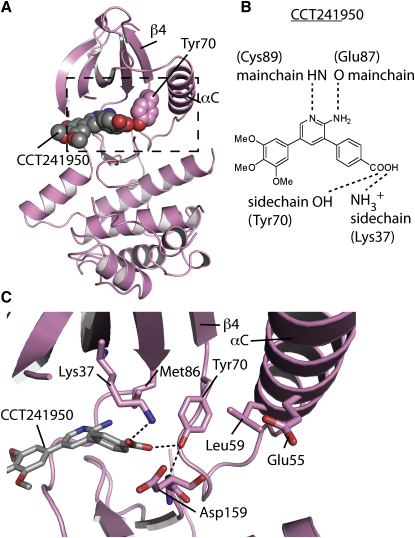
Crystal Structure of Nek2 Bound to a Drug-like Molecule that Induces the Tyr-Down Conformation (A) Nek2-CCT241950 structure shown in cartoon representation in the equivalent view to that shown in Figure 1B. CCT241950 and Tyr70^Nek2^ are shown as spheres. The dashed box indicates the region magnified in (C). (B) Chemical structure of CCT241950 with potential H-bonds marked as dashed lines. (C) Cartoon and stick representation of Tyr70^Nek2^ and its surrounding environment. The putative H-bonds that form a network between CCT241950 and the protein are shown as dashed lines.

**Figure 5:**
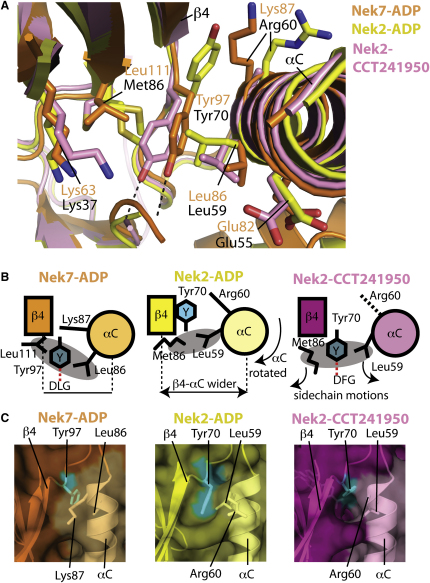
Comparison of Nek7 and Nek2 Structures Reveals the Local Structural Differences between Tyr-Up and Tyr-Down Conformations (A) Superposition of Nek7 (orange) centered on Tyr97 with Nek2-ADP (yellow, PDB code 2W5A) and Nek2-CCT241950 (pink) shows the local structural differences between Tyr-down (Nek7, Nek2-CCT241950) and Tyr-up (Nek2-ADP) conformations. Nek7 residues are labeled in orange, and Nek2 residues are labeled in black. (B) Schematic showing the differences between Nek7 in the Tyr-down conformation, and Nek2 in the Tyr-up and Tyr-down conformations. H-bonds formed by the tyrosine side chain in the Tyr-down conformation are marked with a red dashed line. The tyrosine side chain does not form an H-bond with the protein in the Tyr-up conformation. (C) Surface representation of Nek7-ADP (left, orange), Nek2-ADP (center, yellow), and Nek2-CCT241950 (right, pink) viewed from above αC/β4. The Tyr-down conformation of Nek7-ADP does not create a surface cavity in the same position as that observed in the Nek2-CCT241950 structure. The surface generated by αC helix residues is colored a shade lighter than the surrounding surface.

We have previously described crystals of Nek2-ADP complex, and we have used this system to investigate the binding of small molecules to the ADP-binding pocket (Westwood et al., 2009). CCT241950 induces Tyr70^Nek2^ to jut into the ADP-binding pocket, similar to the Tyr-down conformation of Nek7 (Figure 4A). Tyr70^Nek2^ is the equivalent residue to Tyr97^Nek7^, and in the ADP-bound and other Nek2 structures in the PDB this residue is in the Tyr-up configuration (Figure 5A). CCT241950 is based on an aminopyridine scaffold, which has formed the core of c-Jun and Met inhibitors, although the pattern of H-bonds formed between the scaffold and the Nek2 hinge residues is different from what was observed in the crystal structures of these other two kinases (Figure 4B). The benzoic acid moiety of CCT241950 lies at the center of a network of putative H-bonds that connect the side chains of Lys37 ^Nek2^ and Tyr70 ^Nek2^ and the mainchain of Asp159 ^Nek2^ (Figure 4C). By analogy with the H-bond formed between the DLG motif and Tyr97 of Nek7, this H-bond network is presumably a key determinant of the Tyr-down conformation in Nek2. In support of this assertion, the superposition of the Nek2-ADP and Nek2-CCT241950 structures shows that the conformational changes in the protein primarily involve side-chain movements of the residues that participate in the H-bond network (Figures 5A and 5B). The other major change involves the movement of Met86^Nek2^ and Leu59^Nek2^ side chains to create a pocket into which the side chain of Tyr70^Nek2^ inserts. In the Nek2-ADP structure, the closest contact between Met86^Nek2^ and Leu59^Nek2^ is 4.2 Å, whereas in the Nek2-CCT241950 structure it is 7.6 Å. The Met86^Nek2^ gatekeeper, which is flexible and adopts two rotamers in the ADP-bound structure, moves toward the compound, and the sulfur atom packs against the aromatic ring of the CCT241950 benzoic acid group. Overall there are no substantial changes in the backbone (0.36 Å C_α_ rmsd), although part of the Gly-rich loop and the activation loop are disordered in the CCT241950 structure, perhaps reflecting the loss of stabilizing interactions between the phosphate groups of ADP and the Gly-rich loop and the changes to the activation loop conformation induced through its interaction with CCT241950 and Tyr70^Nek2^. There are no substantial backbone changes (i.e., >1 Å C _α_ rmsd) in the vicinity of Tyr70^Nek2^, which could be because the conformation is constrained by the crystal lattice. Nonetheless, the structure demonstrates that Nek2 can adopt the Tyr-down conformation, with relatively small changes in the overall structure.

We compared the structures of Nek7 and Nek2 Tyr-up/Tyr-down conformations to gain insights into the conserved features and implications for structural mechanisms of regulation (Figure 5). Two conserved features of the Tyr-down conformation stand out: first, the hydrophobic pocket between the gatekeeper residue and the conserved hydrophobic residue on αC (Leu111^Nek7^/Met86^Nek2^ and Leu86 ^Nek7^/Leu59 ^Nek2^, respectively), into which the tyrosine side-chain inserts; and second, the formation of an H-bond between the Tyr side chain and the DFG/DLG motif. Further analysis suggests that side-chain movements would be insufficient to convert Nek7 to a Tyr-up conformation, in contrast to the situation in Nek2. Strikingly, there is no obvious surface cavity for the side chain of Tyr97^Nek7^ to fit into between β4 and αC of Nek7, whereas in the Nek2-CCT241950 structure, the surface cavity is obvious (Figure 5C). Small differences in the region surrounding the tyrosine residue account for the lack of a cavity on the surface of Nek7, which is occupied by the side chain of Lys87^Nek7^ from αC and the backbone of β4 residues 98 and 99 (Figure 5). Relative to Nek2 or Plk1, β4 and the αC of Nek7 are approximately 1 Å closer together, and the αC is rotated and distorted so that Lys87^Nek7^ lies on the surface, whereas Arg60^Nek2^ protrudes from the surface or is disordered. Therefore, the conformational change required to move from the Tyr-down to the Tyr-up conformation in Nek7 is more substantial than just the rotation of the Tyr side chain and would require a concerted set of movements around β4 and αC. We predict that binding of Nek9-CTD to Nek7 remodels these structural elements, creating a pocket on the surface for the side chain of Tyr97.

### Role of the N-Terminal Extensions in Nek7 Activity

**Figure 6:**
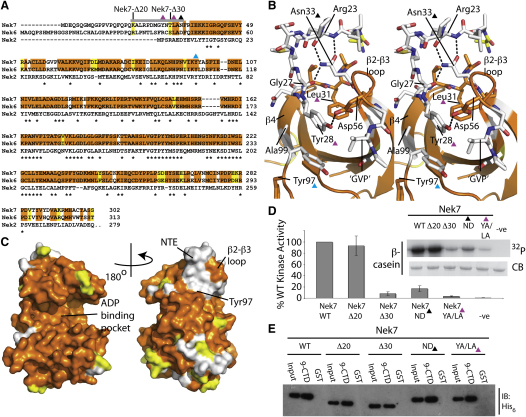
Nek7 Encodes an Unusual NTE Motif that Is Required for Normal Kinase Activity (A) Sequence alignment of human Nek7, Nek6, and Nek2. Sequence conservation between Nek7 and Nek6 is highlighted in orange (identical), yellow (conservative substitution), and white (non-conserved). Residues that are identical in Nek2 are marked with asterisks. The ordered Nek7 NTE is marked with a gray box. Starting residues of Nek7 N-terminally truncated proteins are marked with black bars. Residues mutated in this study are marked with colored triangles: Tyr28^Nek7^ and Leu31^Nek7^, magenta; Asn33^Nek7^, black; Tyr97^Nek7^, cyan. (B) Stereo pair diagram depicts the unusual NTE motif in cartoon and sticks colored by conservation using the color scheme defined in (A). Residues mutated in this study are marked with colored triangles. (C) Sequence conservation between Nek7 and Nek6 mapped onto the surface of Nek7 using the color scheme defined in (A) in two views related by 180°. (D) In vitro kinase activity assay shows the relative activity of wild-type Nek7 (WT), the N-terminal truncations (Δ20, Δ30), and the NTE mutations N33D (ND) and Y28A/L31A (YA/LA). The upper panel shows the autoradiograph (^32^P) of a CB-stained gel in a single assay. The histograms in the lower panel show the average of three independent in vitro kinase assay experiments, and the error bars show the standard deviation. (E) Anti-His_6_ immunoblot of GST coprecipitation experiments using GST-tagged 9-CTD and GST control proteins to capture wild-type and mutant His_6_-tagged Nek7 proteins.

Nek6 and Nek7 are 86% identical within their catalytic domains but are highly divergent in their N-terminal extensions (Figure 6A). The N-terminal extension to the catalytic domain of Nek7 (NTE, residues 20–33) adopts a highly unusual structure, an extended ordered loop wrapped around the side chain of Asn33 (Figure 6B). This side chain forms three H-bonds with the NTE backbone: the backbone oxygen of Gly27 and the backbone oxygen of Arg23 form H-bonds with the Asn33 side-chain nitrogen, and the backbone nitrogen of Arg23 forms an H-bond with the Asn33 side-chain oxygen. Substitution of Asn33 for any other amino acid would disrupt this network of H-bonds and destabilize the local structure. Notably, the equivalent position in Nek6 is an aspartic acid (Figures 6A and 6B). Mapping the sequence conservation between Nek6 and Nek7 onto the structure of Nek7 shows that the two kinases share an almost completely identical front face (Figure 6C, left), with most of the differences lying on the back face (Figure 6C, right). The large patch of nonconserved residues on the N lobe includes the ordered NTE of Nek7 and residues on the core catalytic domain that contact the NTE. The NTE of Nek7 sits on the core catalytic domain on a largely hydrophobic surface between β4 and the β2-β3 loop (Figure 6B). The differences between Nek6 and Nek7 in this region are hydrophilic for hydrophobic substitutions, suggesting that this region of Nek6 may be solvent exposed. For example the sequence “GVP” on β3 of Nek7 is replaced by “RKT” in Nek6, and Ala99^Nek7^ is replaced by aspartic acid. These changes, and the presence of aspartic acid at the equivalent position to Asn33^Nek7^, suggest that Nek6 does not have an NTE-like motif similar to that found in Nek7.

To investigate the function of the Nek7 NTE, we made truncations of Nek7 starting at residues 20 (Nek7-Δ20) and 30 (Nek7-Δ30), a single point mutation of Asn33 to aspartic acid (Nek7-N33D) to disrupt the NTE and a double point mutation to disrupt the interaction of the NTE with the core catalytic domain (Nek7-Y28A/L31A) (Figures 6A and 6B). The proteins exhibited dramatically reduced catalytic activity, with the exception of Nek7-Δ20 (Figure 6D). This was a surprise, because none of the mutations were in the core catalytic domain. We determined the thermal stability of the wild-type and mutant Nek7 proteins by thermal shift assay, a method that measures the increase in fluorescence of a dye that binds to exposed hydrophobic residues upon heating (Figure S2). Full-length, wild-type Nek7 and Nek7-Δ20 have very similar apparent T_m_s of 48.2°C ± 0.3°C and 48.7°C ± 0.1°C. By comparison, the NTE mutant proteins Nek7-Δ30, Nek7-N33D, and Nek7-Y28A/L31A were destabilized with apparent T_m_s of 41.2°C ± 0.2°C, 39.8°C ± 0.2°C, and 36.9°C ± 0.9°C. We conclude that the Nek7 NTE is a structural component of the catalytic domain and thus contributes to activity. The NTE is not required for the interaction with Nek9-CTD, because all truncations and mutations of Nek7 tested bind equally well (Figure 6E). Nek9-CTD also activates Nek6, and therefore Nek9-CTD must bind to a conserved site on the Nek6/7 surface. The NTE is functionally equivalent to the extensions to the catalytic domain that are required for stability in other kinases. The archetype of this feature is in the AGC family of kinases, which possess a C-terminal hydrophobic motif that binds between αC and β4. This is not quite the same site of interaction as the NTE, which sits on the other side of β4, but it is clear that it can fulfill a similar role in stabilization of the catalytic domain. In the case of PKA, the hydrophobic motif is a stable component of the catalytic domain, whereas in the case of PKB the interaction is regulated by phosphorylation (Gold et al., 2006). The NTE of Nek7 appears to be a stable component of the catalytic domain, like the hydrophobic motif of PKA, but we cannot rule out the possibility that it could be regulated.

## Discussion

In addition to the many examples of kinases regulated by phosphorylation, some kinases are regulated through binding of partner proteins. The archetypes of this form of regulation are the family of Cdks, which are activated by Cyclins and inhibited by p16 and p21 (Pavletich, 1999). Other examples of kinases activated by partner proteins include Aurora-A, Aurora-B, and Bud32 (Bayliss et al., 2003; Mao et al., 2008; Sessa et al., 2005). We have now shown that the noncatalytic, CTD of Nek9 directly stimulates the activity of Nek7 through release of Tyr97 autoinhibition. This presumably occurs through a local remodeling of the structure in the vicinity of β4/αC and Nek6 is probably activated in a similar fashion. Nek9 has also been proposed to be the upstream kinase that phosphorylates Nek6 and Nek7 and is therefore capable of stimulating Nek6 or Nek7 activity through two distinct mechanisms. It is not yet clear whether the two mechanisms act in tandem, in sequence, or indeed have distinct functions, although it is known that the interaction between Nek6 and Nek9 is confined to mitosis, and this probably extends to the Nek7/Nek9 interaction (Rapley et al., 2008). Further details regarding the regulation of this network are needed before we can propose a complete model for Nek6 and Nek7 regulation. Crucially, the mechanisms that activate Nek9 have not yet been identified, and neither have the regulatory events that lead to the association of the three kinases. In light of this shortfall, what do our results imply concerning the role of Tyr97^Nek7^/Tyr108^Nek6^ and Nek9 in proper cell-cycle regulation of Nek6 and Nek7? Our results arguably reveal more about the nature of repression during interphase than the mechanism of activation in mitosis, because the Nek7-Y97A and Nek6-Y108A were more active than the wild-type kinases during interphase, not during mitosis. From this we conclude that Tyr-down autoinhibition restricts the activity of Nek6 and Nek7 in interphase but is completely released in mitosis. Excessive Nek7 activity in interphase is clearly deleterious for the cell, and it therefore makes sense that Nek7 has evolved with a highly stringent mechanism to repress its activity and perhaps tether the activity to Nek9. There are no known substrates for Nek7, and so it is not clear which phosphorylation events produce abnormalities in cells expressing Nek7-Y97A. We suggest that constitutively active Nek6/7-YA mutants will be very helpful for the identification of their substrates and in the more precise definition of their functions.

Tyr97^Nek7^ and Tyr108^Nek6^ are clearly important auto-inhibitory features, but could the same mode of regulation be found in other kinases? We have shown that Nek2 can adopt the Tyr-down conformation when bound to a drug-like molecule, although we have not yet established that the Tyr-down conformation plays a regulatory role in Nek2. Other structures of the Nek2 catalytic domain, including the ADP-bound structure, exhibit a Tyr-up conformation. Thus, the catalytic domain of Nek2 does not seem to be intrinsically autoinhibited by Tyr70 but could be regulated through interactions with the CTD, which are absent in the crystallized Nek2, or with another binding partner such as the inhibitory protein HEF1 (Pugacheva and Golemis, 2005). Predicting the possibility of other kinases adopting the Tyr-down conformation depends on whether it is necessary for this residue to be a tyrosine for inhibition and whether there are any unique features of Nek2, -6, and -7 that confer the propensity to adopt the Tyr-down conformation. The series of mutants we made in Nek7 show a clear trend in activity and thus provide some illumination on the first point. The most active mutation, Nek7-Y97A, breaks the H-bond to the DLG motif and removes the hydrophobic block between αC (Leu86^Nek7^) and the gatekeeper residue (Leu111^Nek7^). The next most active mutants, Nek7-Y97F and Nek7-Y97L, break the H-bond but do not remove the hydrophobic block. Therefore the H-bond and the hydrophobic block both contribute to inhibition, and so efficient inhibition at this position requires an amino acid that is hydrophobic and able to form an H-bond with the DFG/DLG motif. Of the amino acids that combine these properties, only tyrosine is commonly found at this position, in over 10% of human kinases, whereas there are only single instances of lysine, glutamic acid, and glutamine. The other residues involved in the Tyr-down conformation of Nek7 and Nek2 are highly conserved among all kinase catalytic domains, namely the hydrophobic αC residue, the hydrophobic gatekeeper residue, and the DFG/DLG motif. The only other example of the Tyr-down conformation in the PDB is in one chain out of four of OSR1. In this chain, αC is partially disordered, Tyr78^OSR1^ packs against the Met92^OSR1^ gatekeeper residue, and the hydroxyl is well placed to form an H-bond with the DFG motif at Gly166^OSR1^, a similar set of interactions to that observed in Nek7. In the other three chains of OSR1, αC is well ordered, and the bulky side chain of Met67^OSR1^ sterically hinders the Tyr-down conformation. The size of the gatekeeper and αC hydrophobic residues may be an important determinant of the propensity to form the Tyr-down conformation, because they form the hydrophobic pocket into which the tyrosine side chain fits. In Nek7 both residues are leucine, in Nek2 the gatekeeper is methionine and the αC residue is leucine, and in OSR1 both residues are methionine. A survey of the other known structures of kinases that exhibit a residue equivalent to Tyr97^Nek7^ shows that most are of phosphorylated, active kinases that could not be expected to show features relevant to the inactive conformation. The other structures exhibit either a steric blockage to the Tyr-down conformation or a hydrophilic gatekeeper that would not be expected to promote a hydrophobic interaction with a tyrosine side chain. It would be no surprise to find other kinases regulated by switching between the Tyr-up and Tyr-down conformations, and these could be identified by mutation of the tyrosine residue. Some kinases could, like Nek7, be intrinsically autoinhibited and the inhibition released through binding of an activating partner protein. In other cases the Tyr-down conformation could be induced through binding of an inhibitory partner protein, perhaps through binding to the β4/αC region.

Mitotic kinases, in particular the Aurora family and Plk1, have generated a lot of excitement in the cancer drug discovery field. Mitotic Neks are candidates to be considered in the next wave of targets because Nek2, -6, and -7 overexpression is associated with several cancers (Capra et al., 2006; Hayward et al., 2004). The complete conservation in the ATP-binding pocket suggests that it will prove difficult to produce an inhibitor that can discriminate between Nek6 and Nek7, but this may not prove to be a drawback, because the two kinases lie in the same pathway and their RNAi knockdowns exhibit very similar phenotypes (O'Regan and Fry, 2009). The high-resolution crystal structures we have solved will provide the basis for a structure-based drug design approach to stimulate the production of a small molecule Nek6 and Nek7 inhibitor. Inhibitors that target an inactive kinase conformation are attractive because only a subset of kinases can readily adopt the specific conformation induced by the inhibitor, and hence the compounds tend to be more selective. The Tyr-down conformation of Nek2 and Nek7 is an inactive conformation that is not accessible to the vast majority of human kinases. This is potentially a good starting point from which to design selective inhibitors that target these kinases, and perhaps others that are able to adopt this conformation, based on standard type I compounds modified using the design principles characterized in the crystal structure of Nek2-CCT241950.

## Experimental Procedures

### DNA Manipulation, Protein Expression, and Purification

Human Nek7 was cloned into pET30 (Novagen) to produce the full-length protein with an uncleavable C-terminal His_6_ tag and was coexpressed with λ phosphatase overnight in Codonplus RPIL (Stratagene) *E. coli* at 18°C following induction with 1 mM IPTG. Cells were lysed by sonication, clarified, and Nek7 protein initially purified using a 5 ml HisTRAP column (GE Healthcare) in 50 mM HEPES (pH 7.5), 300 mM NaCl, 5% glycerol, and a 20–250 mM imidazole gradient. For use in crystallization, Nek7 protein was purified to homogeneity by gel filtration using a Superdex 200 16/60 column equilibrated in 20 mM HEPES (pH 7.5), 200 mM NaCl, 5 mM β-mercaptoethanol, and 5% glycerol. For use in kinase assays, protein was instead dialysed into the gel filtration buffer. Site-specific mutagenesis used the QuikChange method (Stratagene). Mutations were confirmed by DNA sequencing. The CTD of human Nek9 (amino acids 706–960) was cloned into pGEX-4T1. Protein expression in *E. coli* Rosetta DE3 was induced by addition of 1 mM IPTG followed by incubation overnight at 18°C, and proteins were purified using glutathione Sepharose chromatography according to the manufacturer's instructions (GE Healthcare).

### Protein Crystallization

Nek7 was crystallized using the hanging drop, vapor diffusion method. The well buffer composition was 100 mM HEPES (pH 7.5), 15% PEG 5000MME, 400 mM (NH_4_)_2_SO_4_. Drop composition was Nek7 protein at 440 μM mixed 1:1 with well buffer. Crystals were briefly soaked in well buffer supplemented with 20% ethylene glycol and flash frozen in liquid nitrogen. To obtain ADP-bound Nek7, apo crystals were soaked for 24 hr in well buffer supplemented with 2 mM ADP and 2 mM MgCl_2_. Nek2-ADP crystals were prepared as previously described (Westwood et al., 2009), transferred to 3 mM CCT241950 in Nek2 well buffer supplemented with 10% DMSO for 72 hr, transferred to cryoprotectant, and flash frozen.

### Crystallography

Diffraction data were collected at DIAMOND light source, Didcot, UK, at beamlines I04 (Nek7-apo) and I02 (Nek7-ADP and Nek2-CCT241950). Diffraction data were reduced using MOSFLM and scaled using SCALA (CCP4, 1994). Nek7 structure solution used Nek2-ATPγS (PDB code 2W5B; Westwood et al., 2009) as a model for molecular replacement using PHASER searching for N- and C lobes independently. Initial model was built using Arp-WARP, and subsequent refinement and model building used iterative cycles of PHENIX and COOT (Adams et al., 2002; CCP4, 1994). Four TLS groups (residues 20–117, 118–203, 204–254, 255–300) were defined using TLSMD (Painter and Merritt, 2006). CCT241950 refinement (.cif) file was made using PHENIX and modified to restrain the carboxylic acid group to a plane. COOT and PHENIX were used for model rebuilding and refinement of the Nek2-CCT241950 structure. Structural figures were prepared using PyMOL (DeLano, 2002). Sequence analysis used the published human kinome sequence alignment (Manning et al., 2002).

### Cell Culture and Synchronization

HEK293 cells were grown in Dulbecco's modified Eagle's medium (DMEM, Invitrogen) supplemented with 10% heat-inactivated fetal bovine serum (FBS), 100 IU/ml penicillin, and 100 μg/ml streptomycin at 37°C in a 5% CO atmosphere. M phase-arrested cells were prepared by 16 hr treatment with 50 ng/ml nocodazole; rounded mitotic cells were collected by gently pipetting off the floating population. G1/S cells were prepared by 16 hr incubation with 1 mM hydroxyurea prior to lysis. Transient transfections were performed with Lipofectamine 2000 reagent (Invitrogen) according to the manufacturer's instructions.

### In Vitro Kinase Assays

For the assays described in Figures 2 and 6, bacterially expressed and purified kinases were preincubated in kinase buffer (50 mM HEPES.KOH [pH 7.4], 5 mM MnCl_2_, 5 mM β-glycerophosphate, 5 mM NaF, 4 μM ATP, 1 mM dithiothreitol [DTT]) with β-casein as substrate and GST, GST-Nek9-CTD, or no additional protein for 30 min at 30°C. Following this preincubation, unlabeled and radiolabeled ATP were added to the reaction and phosphorylation allowed to proceed for 30 min at 30°C prior to analysis by SDS-PAGE, Coomassie blue staining, and autoradiography. The amount of ^32^P incorporated into β-casein was determined by scintillation counting. For the determination of cell-cycle-specific kinase activities in Figure 1D, lysates were prepared from 293 cells that were untransfected or transfected with Flag-Nek6/7-WT/YA before synchronization in G1/S or M phase of the cell cycle. Anti-Flag immunoprecipitates were used in kinase assays (as above) and subjected to immunoblotting with Flag antibodies.

For the assays described in Figure 3, bacterially expressed and purified kinases were incubated with 2 μCi ^33^P ATP in 38 μM ATP for 50 min at 30°C using β-casein as substrate. The assay buffer used was 20 mM Tris 7.5, 25 mM NaCl, 0.01% Tween 20, 1 mM MgCl_2_, 1 mM DTT. Proteins were separated by SDS-PAGE, transferred to nitrocellulose, and visualized using a Typhoon phosphoimager (Amersham). Activity was quantitated using ImageQuant software (Amersham) and normalized to Nek7-WT activity.

### GST Coprecipitation Assays

GST-fusion proteins (20 pmol) and the GST-only control were each immobilized on 30 μl of glutathione Sepharose 4B beads (GE Healthcare) and washed three times in PD buffer (20 mM HEPES [pH 7.5], 150 mM NaCl, 0.05% Tween 20). Beads were incubated with 1 ml of 3.5 μM Nek6/7 protein for 2 hr followed by three 30 min washes in 1 ml PD buffer at 4°C with agitation. Beads were then resuspended in 100 μl of SDS-loading buffer. The eluted protein was diluted 1/100 into SDS-loading buffer, resolved by SDS-PAGE, and visualized by immunoblotting.

### Thermal Stability Assays

Details of this method are in the Supplemental Data.

### Chemical Synthesis

The synthesis of CCT241950 will be described in a future publication.

## Figures and Tables

**Table 1:** Summary of Crystallographic Analysis

Crystals	Nek7-Apo	Nek7-ADP	Nek2-CCT241950
Space group	*I*222	*I*222	*C*2
Lattice constants			
*a* (Å)	46.94	46.83	100.43
*b* (Å)	132.32	133.11	56.84
*c* (Å)	132.37	131.85	73.49

Data Collection			

Resolution range (Å)	66.2–2.10	66.2–2.30	57.8–2.17
(Highest resolution shell)	(2.21–2.10)	(2.42–2.30)	(2.28–2.17)
Unique reflections	23,808 (3,498)	18,761 (2,686)	17,404 (2,525)
Completeness (%)	96.9 (98.5)	99.9 (100.0)	99.7 (99.9)
Multiplicity	3.6 (3.6)	3.8 (3.9)	3.4 (3.4)
R_merge_ (%)	6.6 (27.8)	12.0 (28.2)	8.9 (38.5)
I/σ (I)	12.5 (3.8)	10.7 (3.3)	10.3 (3.4)

Refinement			

Resolution range	10–2.10	44–2.30	57.8–2.17
Number of reflections	22,221	18,172	16,775
R factor (%)	17.4	19.1	18.3
R_free_^a^ (%)	21.6	22.1	23.5
Number of:			
Amino acids	258	259	240
Waters	186	119	110
Rmsd			
Bond lengths (Å)	0.008	0.007	0.016
Bond Angles (°)	1.06	1.04	1.13
Ramachandran outliers	0.0	0.0	0.0
Ramachandran favored	98.0	96.8	98.3

a Free R factor was computed using 5% of the data assigned randomly.

## Accession Numbers

Atomic coordinates and structure factors for apo- and ADP-bound forms of Nek7 and CCT241950-bound Nek2 have been deposited with the Protein Data Bank under ID codes 2WQM, 2WQN, and 2WQO, respectively.

## Supplemental Data

Document S1. Two Figures and Supplemental References
