# Supplementary material for: An Autoinhibitory Tyrosine Motif in the Cell-Cycle-Regulated Nek7 Kinase Is Released through Binding of Nek9
Source: Mol Cell. 2009 Nov 25;36(4):560–70. doi: 10.1016/j.molcel.2009.09.038 (PMC2807034; doi:10.1016/j.molcel.2009.09.038)
Supplement: Document S1. Two Figures and Supplemental References [file mmc1.pdf]

Molecular Cell, Volume *36*

Supplemental Data

An Autoinhibitory Tyrosine Motif in the Cell-Cycle-Regulated Nek7 Kinase Is Released through Binding of Nek9

Mark W. Richards, Laura O'Regan, Corine Mas-Droux, Joelle M.Y. Blot, Jack Cheung, Swen Hoelder, Andrew M. Fry, and Richard Bayliss

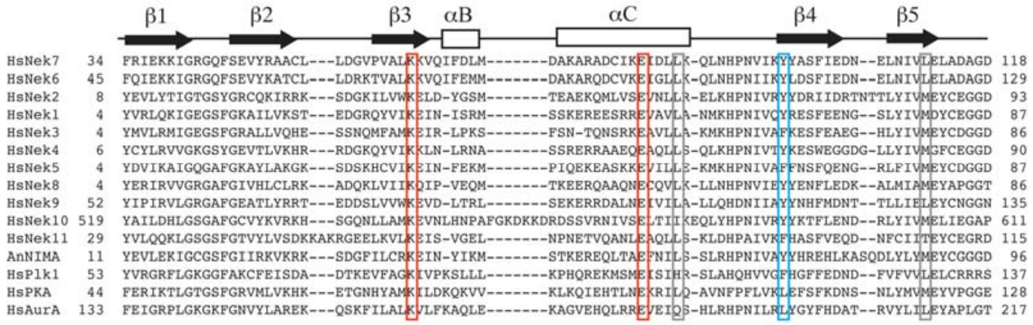

Figure S1. Alignment of catalytic domain N-lobe sequences.
Protein sequences corresponding to the N-lobes from the eleven human Nek kinases (HsNek1-11), *Aspergillus nidulans* NIMA kinase (AnNIMA), human Plk1 (HsPlk1), human Protein Kinase A (HsPKA) and human Aurora-A (HsAurA) were aligned using ClustalW, and manually edited to align the catalytic residues. Residue numbering is shown on the left and right of the sequences. Residues equivalent to the HsNek7 Lys63 and Glu82 are marked with red boxes, Leu111 and Leu86 with grey boxes, and Tyr97 with a cyan box.

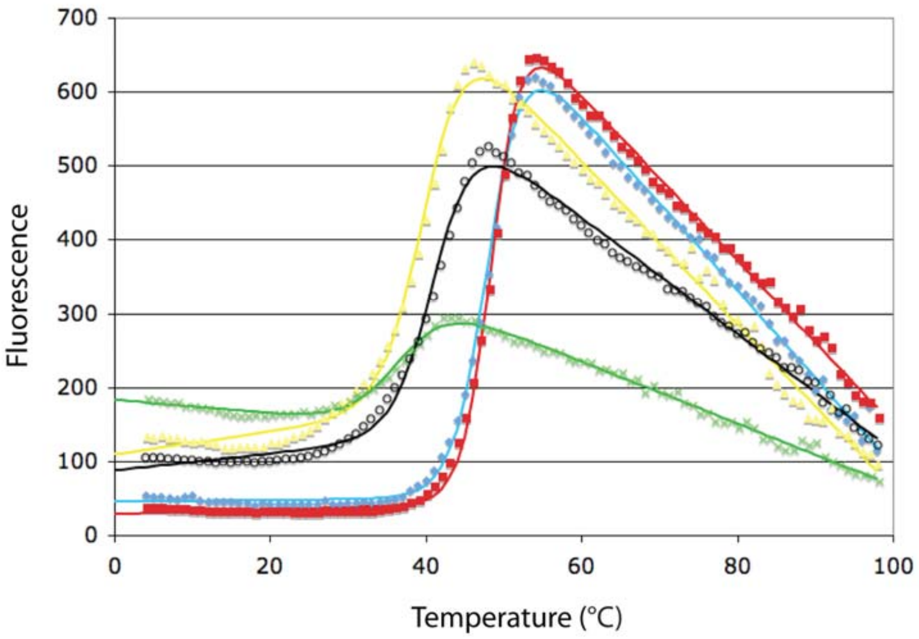

Protein	Apparent T_m (°C)
Nek7	48.2 ± 0.3
Nek7-Δ20	48.7 ± 0.1
Nek7-Δ30	41.2 ± 0.2
Nek7-N33D	39.8 ± 0.2
Nek7-Y28A/L31A	36.9 ± 0.9

Figure S2. Thermal shift denaturation of Nek7.

The graph shows representative thermal shift data (markers) and fitted curves (lines). The elevated fluorescence at low temperature observed for the Nek7-N33D and Nek7-YA/L31A proteins indicates increased exposed hydrophobic surface and/or increased unfolded protein before thermal denaturation. The quoted values are the mean and standard deviation of three repeated experiments.

The procedure for the thermal shift assay was based on a protocol devised by the Structural Genomics Consortium (Niesen et al., 2007). 500 μl of 3.8 μM Nek7 protein samples were prepared on ice in buffer comprising 20 mM Hepes pH 7.5, 200 mM NaCl and 5x Sypro Orange (Sigma). Fluorescence scans were measured using a Varian Cary Eclipse fluorescence spectrophotometer with a Cary temperature controller. Samples were pre-chilled to 4 °C for 10 minutes and then ramped from 4 °C to 98 °C at 2 °C per minute. Sypro Orange fluorescence was excited at 470 nM, and emission measured at 600 nM with 5 nM slits. ProFit software was used to fit data to the equation: $y=(an*x+bn)+((ad*x+bd)-(an*x+bn))/(1+exp((T_m-x)/m))$, where an and ad are the slopes of the native and denatured baselines, bn and bd are the y-intercepts of the native and denatured baselines, T_m is the apparent melting temperature and m describes the slope of the denaturation.

Supplemental References

Niesen, F.H., Berglund, H., and Vedadi, M. (2007). The use of differential scanning fluorimetry to detect ligand interactions that promote protein stability. Nat Protoc *2*, 2212-2221.
